# A framework to improve quality of hospital-based physiotherapy: a design-based research study

**DOI:** 10.1186/s12913-023-09062-x

**Published:** 2023-01-14

**Authors:** Rudi A. Steenbruggen, Marjo J. M. Maas, Thomas J. Hoogeboom, Paul L. P. Brand, Philip J. van der Wees

**Affiliations:** 1 School of Health, Physiotherapy, Saxion University of Applied Sciences, Enschede, the Netherlands, Radboud Institute for Health Sciences, IQ healthcare, Radboud university medical center, Nijmegen, the Netherlands, p/a Saxion, University of Applied Sciences, Postbus 70.000, 7500 KB Enschede, the Netherlands; 2 Institute of Allied Health Studies, HAN University of Applied Sciences, Nijmegen, the Netherlands Nijmegen, the Netherlands, Radboud Institute for Health Sciences, IQ healthcare, Radboud university medical center, Nijmegen, the Netherlands; 3 Radboud Institute for Health Sciences, IQ healthcare, Radboud university medical center, Nijmegen, the Netherlands; 4 Isala Hospital, Zwolle, the Netherlands, University of Groningen and University Medical Centre, Groningen, the Netherlands

**Keywords:** Physiotherapy, Hospital, Quality, Quality improvement, Design-based research

## Abstract

**Background:**

A quality framework for hospital-based physiotherapy is lacking. This study aims to design a framework, building on the currently available literature, to improve the quality of hospital-based physiotherapy.

**Methods:**

A multidisciplinary panel of six representatives of hospital-based physiotherapy and their key stakeholders (patients, medical specialists, hospital management and professional association) was set up. We used brainwriting to sample ideas and the ‘decision-matrix’ to select the best ideas.

**Results:**

The first round of brainwriting with an online panel of six experienced participants yielded consensus on seven possible methods for quality improvement of hospital-based physiotherapy [1]: continuing education [2] ,feedback on patient reported experience measures and patient reported outcome measures [3] ,a quality portfolio [4] ,peer observation and feedback [5] ,360 degree feedback [6] ,a management information system, and [7] intervision with intercollegiate evaluation. Placing these methods in a decision matrix against four criteria (measurability, acceptability, impact, accessibility) resulted in a slight preference for a management information system, with almost equal preference for five other methods immediately thereafter. The least preference was given to a 360-degree feedback.

**Conclusions:**

In the design of a framework for improving the quality of hospital-based physiotherapy, all seven suggested methods were perceived as relevant but differed in terms of advantages and disadvantages. This suggests that, within the framework, a mixture of these methods may be desirable to even out respective advantages and disadvantages.

## Introduction

Hospital-based physiotherapy can play a significant role in the multidisciplinary treatment of hospitalized patients through the optimalization of functional mobility as an important part of the patient’s functional health condition [1]. Good quality treatment is a prerequisite for optimal patient recovery. Quality of hospital-based physiotherapy can be defined as the degree of similarity between criteria of good care (desirable care) and the practice of care (actual care) [2]. In other words, delivering high-quality physiotherapy services in a hospital requires striking a balance between expectations and perceptions of patients and key stakeholders, and to close the gap between the two [3]. To develop a high standard of service quality, a target audience-centred strategy is needed that begins with defining the target audience (patients and key stakeholders) and its needs and wants [4, 5].

In previous research, we identified quality aspects for hospital-based physiotherapy both in the eyes of hospital-based physiotherapists and their key stakeholders: patients, medical specialists, hospital managers, executive boards and co-treating professionals. We also noted that globally expanding accreditation instruments to measure quality such as JCI or Qmentum mainly focus on hospital policy and procedures and do not specifically cover a profession such as hospital-based physiotherapy. These instruments do not allow systematic quality improvement of hospital-based physiotherapy departments [6, 7]. Also, there is no structured system from the national professional association of physiotherapy with suitable means to provide insight into the quality of (departments of) hospital-based physiotherapy. This justifies the need for a tailored quality improvement (QI) framework for hospital-based physiotherapy.

The aim of this study is to gain insight in which QI methods could form the design of a QI framework, as a foundation for a system to improve the quality of hospital-based physiotherapy in the Netherlands, by combining the insights of hospital-based physiotherapists and their key stakeholders. In this context, information from a stakeholder analysis can be used to develop strategies for managing high-quality physiotherapy services for these stakeholders [8, 9]. Ideally, these stakeholders should also be involved in the design, development and selection of measuring instruments for quality improvement [10, 11]. This requires the involvement of all parties, brought together in one room [12, 13]. In this context, design-based research seems to be an appropriate methodology because it allows for iteratively developing, testing and improving innovative QI program designs together with stakeholders. Design-based research contributes towards both testing and refining theories and improving practice and is a fruitful approach for (re-)designing work-based environments and assessment programs [14].

## Method

To comply with the principles of design-based research, we identified relevant stakeholders of hospital-based physiotherapy in QI by conducting a stakeholder analysis [12, 13]. We involved all identified key stakeholders in the design process from the start and set up a panel comprising them: a medical specialist, a hospital manager, a hospital-based physiotherapist, a manager of hospital-based physiotherapy, a patient, and a representative from the quality department of the professional association KNGF (Royal Dutch Society for Physiotherapy). Potential participants needed to have active experience with hospital-based physiotherapy from their respective positions and in participating in representative bodies. It was also predetermined that this group would not suffer from conflict of interests because they operate independently in their day-to-day work. We aimed to include a total of six participants for this panel, who were approached for participation via the authors’ formal and informal networks. Due to COVID-19 restrictions, the panel session was planned online. A week before the panel meeting, the participants received specific information about the nature and goal of the panel meeting. In addition, the panel received information about the quality themes found in previous research (Table 1) [6, 7], together with the request to contemplate about a method to improve the quality of hospital-based physiotherapy based on these themes.

**Table 1:** Quality Themes for hospital-based physiotherapy

Quality Themes Inside-Out	Quality Themes Outside-In
*The department of hospital-based physiotherapy:*	*The quality of hospital-based physiotherapy is characterised by:*
has a culture of continuous learning, improvement and open dialogue	a human approach
ensures the promotion of staff expertise that is consistent with the demand for care	context-specific and up-to-date applicable knowledge and expertise
uses a planning & control cycle to work on achieving its goals in the short, medium and long term, with a policy plan that fits within the frameworks of organisational policy	providing the right care in the right place at the right time
forms an integral part of the overall patient and hospital process	a proactive departmental policy in which the added value for the hospital is transparent
implements a patient-oriented policy	professional development and innovation based on a vision on science and developments in care
systematically ensures that the physiotherapeutic interventions undertaken by its employees are of the highest possible quality	easy access and awareness of one’s own and others’ position within the interdisciplinary cooperation
collects feedback on its performance from stakeholders and staff and takes action that is based on this feedback	ensuring a continuum of care with the inclusion of pre-and post-clinical care of patients

The panel session was moderated by the first two authors [RS, MM]. After an introduction to the background, purpose, and procedure of the meeting, the panel members participated in a brainwriting session, followed by the construction of a decision matrix. According to DESIGN-BASED RESEARCH principles, these methods are the two most appropriate techniques in the initial phase of a design process [12, 15]. Convening and consulting a voluntary expert panel is exempt from medical ethical review under Dutch law. All panel members provided written informed consent.

### Brainwriting

Brainwriting is an idea generation technique in which participants write down their ideas about a particular question for a few minutes without talking. Then, each person passes his or her ideas to the next person who uses them as a trigger for adding or refining their own ideas [12]. We used the 6–3-5 brainwriting method. Each panel member was asked to individually write down 3 ideas about a method to improve the quality of hospital-based physiotherapy, based on the previously identified quality themes (Box 1) [6, 7]. After 5 minutes, each panel member was asked to pass their own form to another panellist so that 6 rounds of idea generation could take place. With each new round, participants were asked to involve or to build on previous panellists’ ideas. Because this was an online session, due to Covid restrictions, we used Padlet [16]. Padlet is an online environment to gather opinions or ideas. During the digital brainwriting sessions, the research question was always visible for the participants to ensure that all panellists worked towards the same goal. After the final round, each participant received their original form in return and was asked to individually identify the best ideas on this form in 10 minutes. These ideas were shared with the panel followed by a half-hour panel discussion, aiming for consensus on the ideas that were perceived sufficiently appropriate to proceed to the next part of the meeting, namely the decision matrix. The panel discussion was video recorded for analysis purposes.

### Decision matrix

To decide which of the remaining ideas from the first part would be the most suitable, we placed each idea in a decision matrix against a set of decision criteria. For this purpose, the panel was first asked to generate ideas for decision criteria, and then to decide by total consensus which of these criteria should be used. After consensus was reached, the matrix form was filled with ideas and criteria, and each panel member was given half an hour to individually test each idea against each criterion. This was done both quantitatively (providing scores on a Likert scale of 1 (very inappropriate) to 5 (very appropriate)) and qualitatively (by writing comments in text boxes). Finally, all panellists sent their form to the moderators and explained their ideas what the design of a framework should look like to the panel. This marked the end of the panel session. All panel discussions were video recorded for analysis purposes.

### Analysis

Quantitative data from the decision matrix were analysed and described using Microsoft Excel. Written qualitative data from the decision matrix were collected and added as comments to the scores. These comments were checked by both moderators against the various video recordings and supplemented if they highlighted new perspectives. This resulted in a final decision matrix. The research team developed a QI framework design by discussing the outcomes of this final decision matrix. The video recordings were also used to check afterwards whether all procedures during the panel session had been conducted correctly.

### Reflexivity

During the study, we were aware of our positions and maintained a reflexive approach from our perspectives as experienced hospital-based physiotherapist and researcher [RS], as a teacher of physiotherapy and experienced researcher [MM] and as (associate) professors in allied health and medical care and experienced researchers [TH,PB,PW]. None of the authors worked as a hospital physiotherapist in any of the hospitals involved or maintained personal contacts with any of the panellists. We tried to obtain balanced data by having RS conduct the panel, supported by MM. To encourage trustworthiness, a member check of the final decision matrix with all participants was carried out.

## Results

The online panel session took place in December 2021 with six participants: a medical specialist (cardiology), a hospital manager (orthopaedics), a hospital-based physiotherapist, a hospital-based physiotherapy department manager, a patient, and a representative of the quality department of the professional association (Royal Dutch Society for Physiotherapy) (Table 2).

**Table 2:** Characteristics of panel

Member	Gender	Experience Years	Type of Hospital	Relationship to hospital-based physiotherapy
Medical Specialist (Cardiology)	Male	7	General Teaching	Referrer to hospital-based physiotherapy
Hospital Manager (Orthopaedics)	Male	24	General Teaching	Former hospital-based physiotherapist managing major referring specialisms
Hospital-based Physiotherapist	Male	14	University	Active hospital-based physiotherapist
Hospital-based Physiotherapy Department Manager	Male	27	University	Active manager of a major academic department of hospital-based physiotherapy
Patient representative	Male	19	University	Experienced as a patient of hospital-based physiotherapy, followed by activities and experience in patient representative bodies.
Representative of professional Association	Female	11	N/A	Policy officer of the Dutch Association of Physiotherapy in Hospitals

The brainwriting session yielded consensus on seven QI methods: (1) continuing education, (2) feedback on PREMs and PROMs, (3) a quality portfolio, (4) peer observation and feedback, (5) 360 degree feedback, (6) a management information system and (7) intervision with intercollegiate evaluation (Table 3).

**Table 3:** Overview of quality improvement methods with perceived advantages and disadvantages

Method	Objective	Construction	Advantages	Disadvantages
**Continuing Education**	To keep professionals up to date on the latest advances in their field and to afford an opportunity to explore other areas in this field	There are many types of continuing education for professionals, individually or in groups, like: post-secondary degree programs, professional certifications, independent studies, professional events, on-the-job training, research and online courses	Useful Acceptable	Difficult to evaluate the impact on QI Available budget can be a bottleneck
**Feedback PREMs and PROMs**	To foster improvement and adopt best practices based on patient related outcomes and experiences, and in addition clinical outcomes, to further improve these outcomes	Reports coming directly from patients about how they feel or function in relation to a health condition and its therapy without interpretation by healthcare professionals or anyone else	Excellent in providing easily accessible data from a (national) database	Confrontation of the individual professional Effort and cost to setup a (national) database
**Quality Portfolio**	To establish a readable classification at a certain level indicating the quality of the professional on the basis of experience and education	Implementation of a database that shows the relevant experience and education received by each professional	Easy to measure Uncovers gaps in knowledge and skills Fast to apply	Knowledge and skills needs to be in good order for proper functioning
**Peer Observation and Feedback**	To observe each other’s practice and learning from one another, to support the sharing of best practice and build awareness about the impact of your own professional conduct	After a predetermined time period and feedback list the observer may share his/her observations, in the form of a written report accompanied by verbal feedback	Promotion of a culture of feedback and dialogue Little costs	Hard to measure (more qualitative than quantitative data) Might be perceived as threatening
**360 Degree Feedback**	To offer employees more varied multidisciplinary input. To give employees timely recognition and a better understanding how they can improve	A process where the employees receive feedback from peers working closely with them - co-workers, managers, direct reports. The feedback is usually anonymous and completely confidential	Multidisciplinary feedback	Unwillingness to critically appraise multidisciplinary colleagues leading to limited reliability
**Management Information System**	To provide information for decision making on planning, initiating, organizing, and controlling and to provide a synergistic organization in the process.	The Management Information System design should give, after determining the input to be fed to the system, reports in line with the organization structure and needed outcomes. In this case specifically on critical indicators for hospital-based physiotherapy.	Data already available in other systems	Hard to establish which critical process indicators should be implemented
**Intervision with intercollegiate Evaluation**	To share problems, questions, concerns with colleagues in order to develop the skills and insights of professionals who try to look for solutions.	A structured method of group consultation. During a meeting one participant is in the centre with a practical situation from his or her work. The participant describes clearly for what aspects he or she wants input (help) from the others	Accepted method Easy to introduce in work routines	Hard to measure (more qualitative than quantitative data) Might be perceived as threatening

At the start of the next round of the decision matrix, an overall consensus was reached on four criteria against which the seven ideas generated would be assessed: measurability (discriminatory power), acceptability (safety and acceptance), impact (focus and efficiency), and accessibility (cost and effort). After all the scores and comments of the participants per possible idea (prototype) and criterion were collected and discussed, the digital panel session was closed. Subsequently, both moderators put all the scores and comments into a comprehensive overview (Table 4).

**Table 4:** Generalised Decision Matrix (quantitative scores in median, qualitative comments: ⊕ = positive, ± = neutral, − = negative, green shaded = highest score on criterion)

***Scores on a scale of 1 to 5:*** ***1 = very inappropriate*** ***2 = inappropriate*** ***3 = sufficiently appropriate*** ***4 = appropriate*** ***5 = very appropriate***	Criterion 1: Measurable (an essential and distinguishing attribute: discriminatory power)	Criterion 2: Acceptable (adequate to satisfy a need, requirement or standard: safety, acceptance)	Criterion 3: Impact (to have a strong effect on quality: focused and efficient)	Criterion 4: Accessible (easy to understand and use: cost, effort)
**Method 1:** **Continuing Education** ***Median Score: 4,0***	**Score: 3** ± Number of courses is measurable, but doubts about discriminatory power - Measurable to what extent someone has taken it, not what someone has learned from it	**Score: 4** ⊕ This will be acceptable for everyone - If you have to make certain development according to departmental plan, possibly not acceptable	**Score: 4** ± If it also concerns non-physiotherapeutic skills, such as PDCA ± Training is the 1st step, implementation/application the 2nd step - This does not give a good impression of the quality (attendance obligation versus result obligation)	**Score: 4** ± Low effort, high cost ± Costs are manageable at team level - Dependent on departmental budget
**Method 2:** **Feedback PREMs and PROMs** ***Median Score: 4,0***	**Score: 5** ⊕ If PREMS and PROMS are collected per person or per department, this can be easily measurable	**Score: 3** ± In team with a “just-culture” acceptable ± Does require guidance and explanation - This can produce confrontational data	**Score: 5** ± This is very focused, gives a good picture. Can take a lot of effort to retrieve this data. ± Easy and targeted, condition is a good set of prems and proms	**Score: 3** ± Does require some effort and decisiveness from a department - Set-up can entail a lot of effort/work and a lot of costs
**Method 3:** **Quality portfolio** ***Median Score: 4,0***	**Score: 5** ⊕ If knowledge and skills for quality portfolio are tested annually, this can be easily measured	**Score: 4** ⊕ When knowledge and skills are in good order, this may not be a problem	**Score: 4** ⊕ Experience shows that this works well and uncovers gaps in knowledge and skills ⊕ Easy and fast to apply	**Score: 3** ⊕ Little effort, little cost
**Method 4:** **Peer Observation & Feedback** ***Median Score: 4,0***	**Score: 4** ⊕ Requires uniform application ± If this is done using rubrics, this can be easily measured - More qualitative by nature	**Score: 3** ⊕ Can also promote a culture of feedback and dialogue ⊕ In team with a “just-culture” acceptable. Also, acceptable if you manage security well (e.g., anonymously) ± Requires explanation and experience - Can be threatening to have a look behind the scenes	**Score: 5** ± Very direct and efficient way ⊕ Peer Feedback is often considered to be very valuable, especially when adding a feedback course ⊕ Easy to use and targeted	**Score: 4** ± Little cost, some effort - Team leader must be the driving force, is a risk for success
**Method 5:** **360 degree feedback** ***Median Score 3,0***	**Score: 3** - The degree to which someone is willing to ask for feedback has a great influence on the result - More qualitative by nature - Requires training, experience: colleagues have difficulties with this	**Score: 3** ± In team with “just-culture” acceptable ± Dependent on free choice in this - Not everyone will find it convenient to collect feedback	**Score: 4** ⊕ Is multidisciplinary feedback (only valuable alongside peer feedback) ± Provided it is performed well - Pleasing each other can distort	**Score: 4** ± Little cost, some effort - Difficult to complete, difficult to ask whom to ask
**Method 6:** **Management Information System** ***Median Score: 4,5***	**Score: 5** ⊕ Pre-eminently measurable matters ⊕ Establish the Critical Process Indicators as a team and include them in the annual development discussion - What are those Critical Process Indicators?	**Score: 4** ⊕ This data is already being collected ± It is a little unclear which Critical Process Indicators are involved; this is a determining factor for this criterion	**Score: 3** ⊕ Agreements are recorded ± Difficult to estimate - Says little about quality	**Score: 5** ⊕ Is already there, no cost, no effort ± Difficult to estimate
**Method 7:** **Intervision with intercollegiate Evaluation** ***Median Score: 4,0***	**Score: 3** ± Whether measurable depends on methodology/score form ± Provided it is carried out properly - More qualitative by nature	**Score: 4** ⊕ Accepted working method ⊕ After some experience - This can be experienced as threatening	**Score: 5** ⊕ Very direct and efficient way	**Score: 4** ⊕ Easy to fit in ± Little cost, but effort

**Fig. 1:**
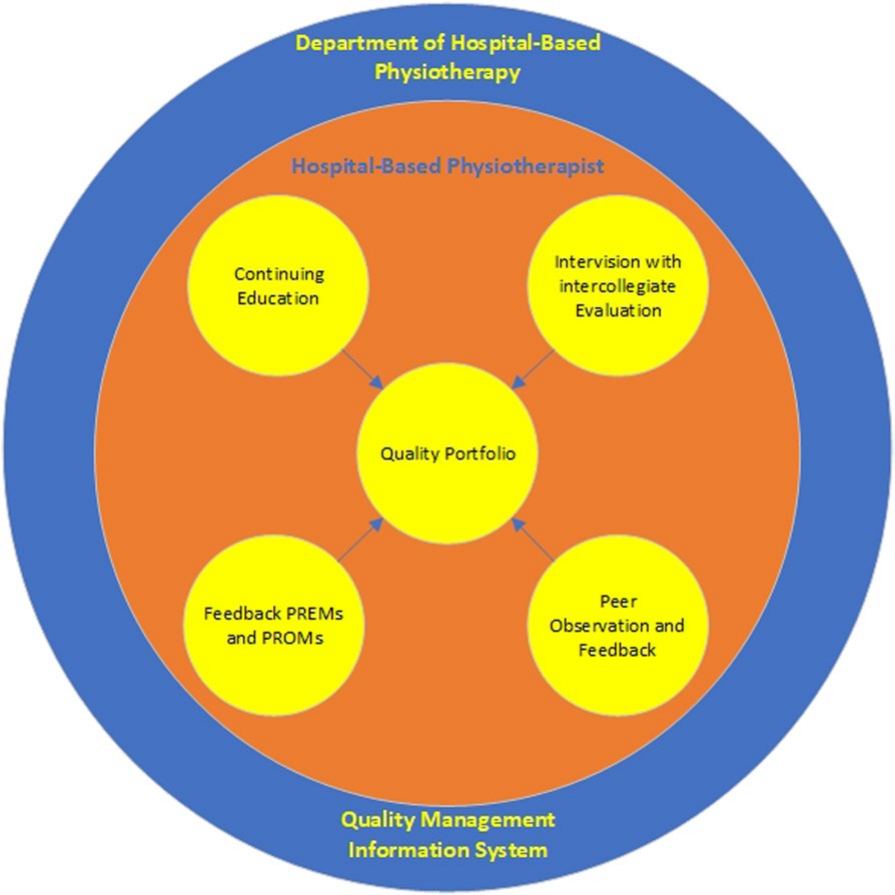
Design of a Framework for Quality of Hospital-Based Physiotherapy

In a member check, all participants agreed individually that this was a correct representation of all that had been discussed and scored. Finally, the result of this study was summarised in the design of a framework for quality of hospital-based physiotherapy (Fig. 1), where the inner circle represents the individual physiotherapist with the factors than can influence individual quality, registered in an individual portfolio. The outer circle represents the department of hospital-based physiotherapy, where overarching quality factors are collected.

### Quantitative data

The median scores of all the criteria per idea ranged from 3.0 (360-degree feedback) to 4.5 (management information system) (Table 4). The median scores of the other five ideas was 4.0. On three of four criteria, the ‘management information system’ idea received highest scores. Feedback on Patient Reported Experience Measures (PREMs) and Patient Reported Outcome Measures (PROMs), a quality portfolio, and intervision & intercollegiate evaluation scored highest on two of four criteria. Continuing education and peer observation and feedback scored the highest on the criterion of acceptability. The idea of 360-degree feedback was not among the highest scores on any criterion.

### Qualitative data

Participants discussed potential advantages and disadvantages of the proposed QI methods, which are summarised in Table 3, together with their objectives and construction:

1. Participants commented that ‘continuing education’ would be an acceptable QI method, but that it would be difficult to evaluate the impact on QI, because it is only measurable to what extent someone has taken a course, not what someone has learned from it. Also, an available budget may be a bottleneck for this QI method.
2. Concerning feedback on PREMs and PROMs, panellists remarked that the measurability of the method is excellent in providing easily accessible data that are sampled and aggregated in a national database, but feedback of patient experiences and outcomes requires guidance and explanation. The setup of this system especially for hospital-based physiotherapy can entail much effort and costs.
3. Comments on a quality portfolio were mainly positive: easy to measure, it uncovers gaps in knowledge and skills and is easy and fast to apply.
4. About peer observation and feedback, participants commented that this QI method provides qualitative rather than quantitative information, that it could be confrontational and threatening to professionals and therefore requires guidance and explanation. But also, this method can promote a culture of feedback and dialogue, works directly and efficiently, and costs little.
5. The positive side of 360-degree feedback was highlighted as a form of multidisciplinary feedback, allowing multiple perspectives on professional performance. As a potential disadvantage, participants commented that the information this QI method provides may not always be reliable due to unwillingness of professionals to critically appraise their multidisciplinary colleagues, possibly resulting in overly positive reports.
6. The general comment on a management information system was that it is hard to establish which quality outcome indicators should be implemented and whether or not this data is already available in other information systems. But once this system is up and running, the advantages are measurability, little cost and no effort.
7. On the idea of intervision and intercollegiate evaluation, participants commented that this is already an accepted direct and efficient working method, which is easily applicable. But also, this is a system more qualitative by nature and can be experienced as confrontational and threatening by professionals.

## Discussion

The goal of this study was to gain insight in which QI methods could form the design of a QI framework, as a foundation for a system to improve the quality of hospital-based physiotherapy in the Netherlands, by combining the insights of hospital-based physiotherapists and their key stakeholders. Out of the seven proposed QI methods, none stood out in ensuring quality improvement. According to the multidisciplinary panel that we consulted, 360-degree feedback was seen as the least suitable QI method and therefore not further exploited as a QI method in this study. Of the other six proposed QI methods, there was a slight preference for a management information system. The panel’s scores and their comments reflected similar appreciation for continuing education, feedback on PREMs and PROMs, a quality portfolio, peer observation and feedback, and intervision with intercollegiate evaluation. The panellists established that each QI method has its own advantages and disadvantages (Table 3).

### Relation to similar studies

The effects and feasibility of each QI method mentioned by the panel have been described previously in the literature. Overall, these studies suggest positive effects and reasonable feasibility, but also make reservations about each method ranging from the degree of effect, reliability, and validity to efforts with and conditions under which application could be successfull [17–28]. The results of these studies suggest that, when designing a QI framework for hospital-based physiotherapy, a mixture of these methods may be most appropriate. This allows evening out of advantages and disadvantages of each individual method, because they cover different aspects of professional quality. The result may be a combination of methods that together meet the predefined QI criteria and build a valid and effective framework to improve the quality of hospital-based physiotherapy.

More rigorous research is needed to identify effective and generalizable interventions individually, but also in combination as a multiple method assessment, to improve healthcare quality [29, 30]. This may lead to a more multidimensional approach to quality [31, 32].

The prevailing method of the Individual Quality Register of Physiotherapy of the KNGF in primary care is individually based, where each activity aimed at professional development is rewarded with points [33]. For hospital-based physiotherapy, an integrated approach based on a portfolio of activities would be more appropriate. This is in view of the nature of the work of hospital-based physiotherapists, which can be more short-cycled, more acute, more varied and more multidisciplinary than in primary care. Especially in a healthcare environment that promotes the collaboration of administrators and physicians in ensuring the quality of patient care [34], a multidimensional model also offers advantages in terms of a more flexible applicability to different disciplines pursuing the same quality goal. Also, because hospital-based physiotherapy is bound to other regulations than in primary care, this flexibility of a multidimensional model offers more options for QI. Especially if techniques that are already used in the hospital world, such as the tracer method with peer observation and feedback, are used [25].

### Meaning and relevance of the findings

The results of this study, summarised in the design of a framework for quality of hospital-based physiotherapy (fig. 1), provides a foundation to develop a quality system for hospital-based physiotherapy. A quality system comprises a management system and a technical system (methods for IQ). Here, the individual professional manages his own quality efforts in a personal portfolio, which is fed by four types of quality improvement methods. These methods each highlight a different aspect of quality so that a total package is created that fits the described nature of work of hospital-based physiotherapy. The management information system concerns all activities in the field of planning, decision-making, organisation, control, evaluation, motivation, training, and involvement of employees to guarantee and improve quality [35]. Within this management information system, quality indicators found in previous research [6, 7] could be implemented.

### Strengths and limitations

The composition of a representative panel for hospital-based physiotherapy enables a balanced answer to our research question. Using the principles of design-based research is another strength, as design-based research studies can play an important role in the advancement of theory and practice in designing or redesigning work-based learning environments and assessment programs [14]. Although exact data on its validity and reliability are still lacking, the method of brainwriting has been presented as a novel and efficient alternative to brainstorming that can rapidly inform program implementation at minimal time and cost [36–38].

We acknowledge the following limitations. Although a design-based research panel can produce collective answers, the achieved consensus is not necessarily accurate; bias can occur in the meeting because one individual’s opinion can be overrepresented. Since the panel meeting was not anonymous, respondents may have felt restrained to speak freely, and may have been subject to social desirability bias, especially considering the high scores that were given to the QI methods. Although the panel represented all key stakeholder groups, there was only one representative for each group in the panel, which may have produced selection bias. Also, gender was not considered in the composition of the panel and thus the formation may not have been sufficiently ‘inclusive’, looking at the diversity of interests and perspectives.

A key limitation is the extent to which the results of this design-based research can be generalised or transferred to other contexts. Seen from the perspective of our design and analysis, we think that extrapolation of our results to the Dutch situation of hospital-based physiotherapy is feasible. From an international perspective, this is more complex because the forces within the health care system differ per country, and the positioning of hospital-based physiotherapy can be quite divergent.

### Suggestions for further research

In the search for the right mix of the various QI methods, further studies should investigate what this could look like in terms of impact and feasibility. Within the framework of hospital-based physiotherapy, the QI methods discussed can be further explored, either individually or in certain combinations. If a suitable combination seems to have been achieved, which feeds into a management information system on QI of hospital-based physiotherapy, a follow-up study can be conducted to examine its feasibility and total effect on quality. The main question then is how to measure this quality, and with which quality indicators.

## Conclusion

In the design of a framework for improving the quality of hospital-based physiotherapy, a suitable single method for QI does not stand out in this study. 360-degree feedback was considered least suitable. From the other six proposed methods (continuing education, feedback on PREMs and PROMs, a quality portfolio, peer observation and feedback, a management information system and intervision with intercollegiate evaluation), a management information system was slightly preferred. Each of these methods has its advantages and disadvantages and cover various dimensions and aspects of quality and quality improvement This indicates that within a QI framework, a mixture of these methods may be desirable so that individual disadvantages of each method can be offset by the advantages of other methods.

## Data Availability

The datasets used and/or analysed during the current study are available from the corresponding author on reasonable request.
